# Development of type 1 diabetes mellitus after nivolumab dose escalation: A case report

**DOI:** 10.1097/MD.0000000000043356

**Published:** 2025-07-18

**Authors:** Sayaka Mabuchi, Naoko Adachi, Atsushi Nagasawa, Satoshi Nabika

**Affiliations:** a Department of Human Resources Development for General Practitioner, Shimane Prefectural Central Hospital, Izumo, Japan; b Department of Endocrinology, Shimane Prefectural Central Hospital, Izumo, Japan.

**Keywords:** immune checkpoint inhibitor-induced type 1 diabetes mellitus, immune checkpoint inhibitors, immune-related adverse events, nivolumab, programmed cell death-1 antibodies

## Abstract

**Rationale:**

Immune checkpoint inhibitor–induced type 1 diabetes mellitus (ICI-T1DM) is a rare but serious immune-related adverse event associated with programmed cell death-1 inhibitors such as nivolumab. While previous reports have documented its occurrence, the relationship between nivolumab dosing and the onset of ICI-T1DM remains unclear. This study presents a case of ICI-T1DM following a nivolumab dose and includes a literature review.

**Patient concerns:**

A man in his 50s (weight: 49.4 kg, body mass index: 17.65 kg/m^2^) with advanced esophageal cancer had been receiving nivolumab (240 mg every 14 days) for over 2 years without adverse effects. Because of treatment adjustments, the dose was increased to 480 mg and administered every 28 days. Ninety-six days after the dose increase, he developed acute-onset fatigue, anorexia, and thirst.

**Diagnoses:**

Laboratory tests confirmed diabetic ketoacidosis with hyperglycemia (582 mg/dL), low C-peptide levels, and negative islet-associated antibodies, leading to the diagnosis of nivolumab-induced fulminant type 1 diabetes mellitus.

**Interventions:**

The patient was treated with insulin and discharged after stabilization.

**Outcomes:**

This case suggests that higher dose nivolumab may increase the risk of ICI-T1DM, especially in low body-weight individuals.

**Lessons:**

Given that nivolumab remains effective at lower doses, dose optimization may help mitigate immune-related adverse events while maintaining therapeutic efficacy.

## 1. Introduction

Programmed cell death-1 (PD-1) antibodies are immune checkpoint inhibitors (ICIs). Blocking anti-PD-1 restores pro-activating signaling, resulting in effective antitumor T-lymphocyte responses. Among these, nivolumab has been widely adopted for various malignancies. The use of ICIs has increased globally. In the United States, Medicare expenditure on ICIs rose by 1916% in the period from 2014 to 2019, from $285,506,498 to $5,755,319,571. Concurrently, overall Medicare Part B drug expenditure increased by 57%, from $23,679,547,748 to $37,271,080,631. Expenditure on ICIs thus accounted for 40% of the total increase in Medicare Part B drug spending over this time period.^[1]^ This global rise in ICI (PD-1 antibodies) use has led to greater recognition of immune-related adverse events (irAEs), including rare but serious endocrinopathies such as ICI-induced type 1 diabetes mellitus (ICI-T1DM).^[2]^

ICI-T1DM is a known irAE associated with PD-1 inhibitors like nivolumab. According to the safety database created by Ono Pharmaceutical Company and Bristol–Myers Squibb, of 20,600 patients treated with nivolumab in Japan between July 4, 2014 and August 15, 2017, 67 patients (0.33%) developed type 1 diabetes or fulminant type 1 diabetes.^[3]^ The mean time to onset (i.e., duration between the dates of the first anti-PD-1 antibody injection and the development of type 1 diabetes) was 155 days (range: 13–504 days).^[3]^ While previous studies describe 2 risk factors of ICI-T1DM, namely pre-existing type 2 diabetes (odds ratio [OR], 5.91; 95% CI, 3.34–10.45) and combination ICI therapy (OR, 2.57; 95% CI, 1.44–4.59),^[4]^ the patient in the current case study had no personal or family history of diabetes, he developed fulminant T1DM only after the nivolumab dose was increased to 480 mg, despite remaining asymptomatic on 240 mg for over 20 months, and the onset occurred relatively rapidly (96 days) after the dose increase, whereas previously reported cases had a broader range of onset (almost cases of ICI-T1DM occurred less than 1 year after starting nivolumab therapy), typically unrelated to dose. To our knowledge, this is the first reported case suggesting a temporal association between nivolumab dose and onset of ICI-T1DM in a low body weight patient. This observation may have implications for dose optimization to mitigate irAE risk while preserving therapeutic benefit. Herein, we describe this case of ICI-T1DM after increasing the nivolumab dose and review the relevant literature to contextualize this case and highlight potential implications for dose optimization.

## 2. Case presentation

The patient was a man in his 50s who presented with fatigue, anorexia, and thirst. He had no family history of diabetes mellitus. His medical history included allergic rhinitis, for which he was daily taking carbocisteine 1500 mg, montelukast 10 mg, bilastine 20 mg, and fluticasone furoate spray. The patient had been diagnosed with advanced esophageal cancer (Mt cT2(MP)N1M0 cStageⅡ) and had undergone chemoradiotherapy with 5-fluorouracil and cisplatin. Due to disease progression, systemic therapy with nivolumab was initiated 9 months after the start of chemoradiotherapy, at a dose of 240 mg intravenously (i.v.) every 14 days. Despite this treatment, the esophageal cancer progressed, and he subsequently underwent video-assisted thoracoscopic esophagectomy with gastric conduit reconstruction. Forty days after surgery, 240 mg of intravenous nivolumab was restarted every 14 days. After 174 days of resumed nivolumab at 240 mg, the regimen was changed to 480 mg every 28 days for patient convenience, as he continued to work and preferred less frequent dosing. Ninety-six days after switching to a 480 mg every-28-day regimen, he experienced sudden-onset fatigue, anorexia, and thirst and was unable to eat. The patient presented to our hospital with ketosis and a blood glucose level of 582 mg/dL. His random blood glucose levels had ranged from 87 to 168 mg/dL 3 months earlier. Given the clinical presentation, diabetic ketoacidosis based on nivolumab-related ICI-T1DM was suspected.

On admission, his height, body weight, body mass index (BMI), and temperature were 167.3 cm, 49.4 kg, 17.65 kg/m^2^, and 37.1°C, respectively. Physical examination revealed a midline upper abdominal surgical scar. Laboratory tests revealed the following: serum bicarbonate, 17.5 mmol/L; serum pH, 7.239; elevated anion gap, 19.4; hemoglobin A1c, 8.4%; immunoreactive insulin, 16.4 μU/mL; blood C-peptide immunoreactivity, 0.2 ng/mL; and urine C-peptide immunoreactivity under sensitivity. Ketones were found in the blood and urine. Islet-associated antibodies were negative, with no evidence of acute viral infection (Table 1). Human leukocyte antigen (HLA) typing was as follows: DRB1*09:01:02, DRB1*14:06:01, DQB1*03:01:01, DQB1*3:03:02, and HLA-DPB1 antigen DPB1*05:01:01 (Table 2). Based on clinical findings, fulminant type 1 diabetes mellitus due to nivolumab-related ICI-T1DM was diagnosed.

**Table 1:** Laboratory data on admission.

Parameter	Value	Unit	Normal value
TP	7.2	g/dL	6.6–8.1
Alb	4.5	g/dL	4.1–5.1
T-Bil	0.5	mg/dL	0.4–1.5
AST	17	IU/L	13–30
ALT	17	IU/L	10–42
LDH	140	IU/L	124–222
γ-GTP	62	IU/L	13–64
AMY	36	IU/L	44–132
BUN	26.1	mg/dL	8.0–20.0
Cre	1.34	mg/dL	0.65–1.07
eGFR	45.8	mL/min/BSA	
Na	129.6	mEq/L	138.0–145.0
K	5.7	mEq/L	3.6–4.8
Cl	92.7	mEq/L	101.0–108.0
TG	443	mg/dL	40–234
CRP	0.05	mg/dL	0.00–0.14
BNP	19.6	pg/mL	0.0–18.4
HbA1c	8.4	%	4.9–6.0
Glucose	582	mg/dL	73–109
Fasting IRI	1.5	μU/mL	
Fasting CPR	0.1	ng/mL	0.6–1.8
WBC	6750	/μL	3300–8600
RBC	434 × 10^4^	/μL	435 × 10^4^–555 × 10^4^
Hb	14.2	g/dL	13.7–16.8
PLT	27.8 × 10^4^	/μU	15.8 × 10^4^–34.8 × 10^4^
Anti-GAD antibody	<0.5	U/mL	0.0–4.9
Anti-IA-2 antibody	<0.6	U/mL	0.0-–0.5
Anti-insulin antibody	<0.4	U/mL	0.00–0.39
Ketone	3830	μmol/L	26–122
AcAc	1410	μmol/L	13–69
3-OHBA	2420	μmol/L	0–76
TSH	0.88	μIU/mL	0.38–5.38
FT3	1.55	pg/mL	1.68–3.67
FT4	1.09	ng/mL	0.70–1.48
ACTH	12.0	pg/mL	7.2–63.3
Cortisol	13.8	μg/dL	3.7–19.4
Venous blood gas			
pH	7.239		7.36–7.46
pCO_2_	41.0	mm Hg	34–46
HCO_3_	17.5	mmol/L	24–32
BE	−9.5	mmol/L	−2.5 to 2.5
Anion gap	19.4	mmol/L	
Lactate acid	0.2	ng/mL	0.5–1.6
Herpes simplex IgM	−		0–0.799
Herpes simplex IgG	−		0–1.99
Herpes zoster IgM	−		0–0.799
Herpes zoster IgG	+		0–1.99
CMV IgM	−	IU/mL	
CMV IgG	235	AU/mL	0.0–5.9
EBV IgM	<10		0–9.9
EBV IgG	<10		0–9.9
EBV-EBNA	10		0–9.9
Rubella virus IgM	<0.80		0–0.799
Rubella virus IgG	<2.0		0–1.99
Mumps IgM	<0.80		0–0.799
Mumps IgG	4.0		0–1.99
Urine			
Protein	±		
Glucose	4+		
Ketone	4+		
pH	5.5		
Alb	54.0	mg/d	0.0–29.99
CPR	<2.4	μg/d	20.1–155

3-OHBA = 3-hydroxybutyrate, AcAc = acetoacetate, Alb = albumin, ALT = alanine aminotransferase, AMY = amylase, AST = aspartate aminotransferase, BE = base excess, BNP = brain natriuretic peptide, BUN = blood urea nitrogen, CMV = cytomegalovirus, CPR = C-peptide immunoreactivity, CRE = creatinine, EBNA = EBV nuclear antigen, EBV = Epstein–Barr virus, eGFR = estimated glomerular filtration rate, GAD = glutamic acid decarboxylase, Hb = hemoglobin, HbA1c = glycated hemoglobin, IA-2 = tyrosine phosphatase-related islet antigen 2, IRI = immunoreactive insulin, PLT = platelet, RBC = red blood cell count, T-Bil = total bilirubin, TG = triglyceride, TP = total protein, UA = urine acid, WBC = white blood cell count, γ-GTP = γ-glutamyltranspeptidase.

**Table 2:** HLA typing.

DRB1	09:01:02	14:06:01
DQB1	03:01:01	03:03:02
DPB1	05:01:01	

DPB1 = HLA-DPB1 antigen, DQB1 = HLA-DQB1 antigen, DRB1 = HLA-DRB1 antigen, HLA = human leukocyte antigen.

**Figure 1.**
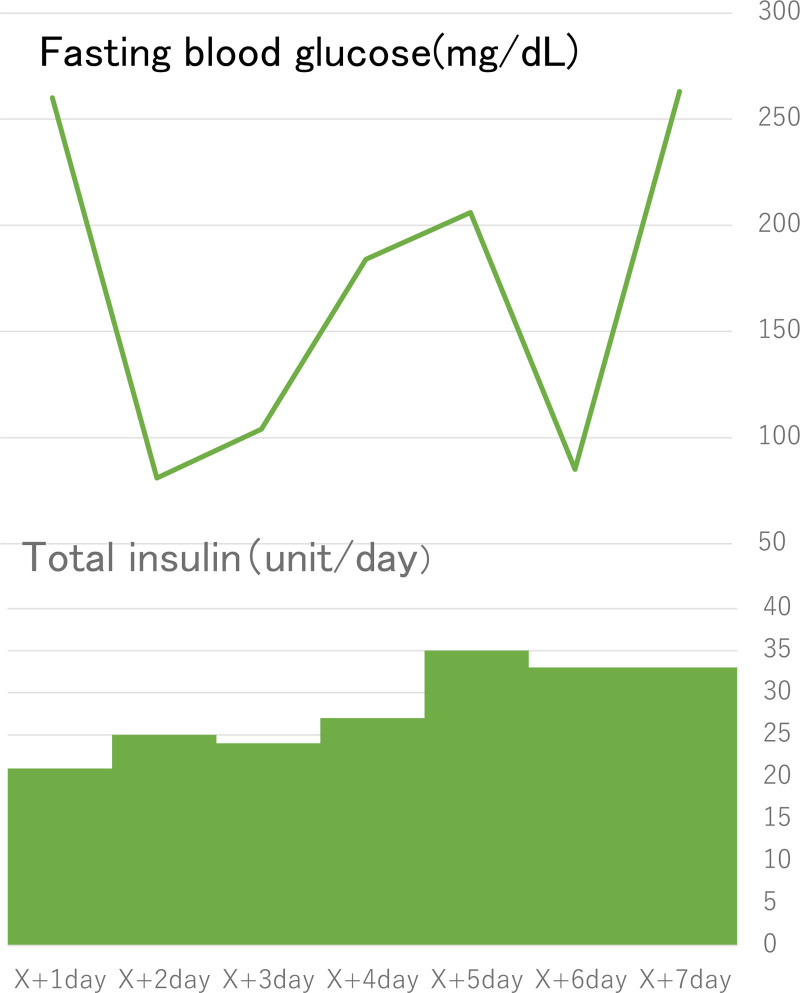
Clinical course and insulin therapy following diagnosis of nivolumab-induced ICI-T1DM. ICI-T1DM = immune checkpoint inhibitor-induced type 1 diabetes mellitus.

The patient was started on continuous venous insulin infusion, which stabilized his blood glucose levels. His treatment regimen was changed from continuous venous insulin infusion to multiple daily injections (total 31 units/d). He was discharged from the hospital 7 days later (Fig. 1). Currently, the patient is doing well. He remains on multiple daily injections (total 34–35 units/d) and uses intermittently scanned continuous glucose monitoring.

This study was approved by the Ethics Review Board of Shimane Prefectural Central Hospital (Approval No. SH24-004). The patient provided informed consent for the publication of this case report.

## 3. Discussion

The patient developed ICI-T1DM following an increase in the nivolumab dose, demonstrating that high-dose nivolumab can cause ICI-T1DM.

Two insights were gained from this case. First, the patient developed ICI-T1DM after the nivolumab dose was increased to 480 mg, despite being asymptomatic for over a year on 240 mg. The median duration between the first anti-PD-1 antibody injection and the onset of nivolumab-related ICI-T1DM was 121 days (range 71–190 days).^[3]^ In this case, the duration was 96 days, which is approximately consistent with the median duration.

Second, given that nivolumab clearance is lower in patients with low body weight, the blood nivolumab concentration may persist at high levels in such individuals.^[5]^ In the current case, the patient’s body weight and body mass index were 49.4 kg and 17.65 kg/m^2^, respectively. Thus, high doses of nivolumab could have persisted in the blood, leading to the development of ICI-T1DM. Lower average nivolumab concentrations may shorten the duration of irAEs by allowing serum nivolumab levels to fall below the toxic threshold more quickly.^[6]^ However, the increased dose may have contributed to the timing of ICI-T1DM onset, in conjunction with other factors that delayed onset until approximately 2 years later. Indeed, some cases have been reported with ICI-T1DM onset occurring more than 1 year after initiation of therapy. Therefore, 3 other factors should be considered regarding the delayed onset of ICI-T1DM.

First, HLA phenotypes should be considered. HLA-DR4 is associated with T1DM, and typical T1DM susceptibility haplotypes are found in 53% of ICI-T1DM cases.^[7–9]^ The median ICI-T1DM onset is significantly delayed in patients with protective HLA-DR alleles (DR7, DR11, and DR15; 18 weeks vs 9 weeks, *P = *.017). In contrast, HLA-DR4 does not significantly affect the timing of ICI-T1DM onset (9 weeks vs 12 weeks; *P* = .696).^[10]^ Since the patient in the current study did not carry protective HLA-DR alleles, the late onset of ICI-T1DM is difficult to explain.

The second point to consider is islet-associated antibodies. Patients positive for any islet-associated antibody tend to develop ICI-T1DM after fewer median treatment cycles than those without autoantibodies (2.5 cycles for those with any positive autoantibody vs 13 cycles for those with negative autoantibodies, *P = *.024).^[11]^ In this case, the patient did not have islet-associated antibodies, making the late-onset of ICI-T1DM again difficult to explain.

Third, other factors that may delay the onset of ICI-T1DM could be identified by analyzing cases where ICI-T1DM occurred more than 1 year after starting nivolumab therapy. In our study, we also reviewed 12 previous such cases^[11–16]^ (Table 3) but found no significant differences in age, sex, or islet-associated antibody levels. Wu et al compared the onset of ICI-T1DM across ICI types (anti-PD-1, anti-programmed cell death ligand 1 [PD-L1]), or combination therapy, including anti-PD-1/anti-PD-L1 with anti-cytotoxic T-lymphocyte activating factor 4, and found no significant association (*P* = .780, *F* = 0.25).^[7]^

**Table 3:** Case reports of immune checkpoint inhibitor-induced type 1 diabetes mellitus more than 1 year after starting nivolumab.

Number	Age/sex	Regimen	Cancer	Duration	Islet-associated antibodies	HLA	Reference
1	80/F	Nivolumab	NSCLC	≥140 wk (20 cycles)	GAD-, IA2-, ZnT8-	NA	Stamatouli et al^[11]^
2	63/M	Nivolumab	RCC	228 wk (78 cycles)	NA	NA	Stamatouli et al^[11]^
3	37/M	Nivolumab + Ipilimumab	Melanoma	418 d	NA	NA	Marsiglio et al^[12]^
4	66/M	Nivolumab 480 mg	Melanoma	363 d	NA	NA	Marsiglio et al^[12]^
5	70/F	Nivolumab 480 mg	Papillary RCC type1	374 d	GAD-, IA2-	NA	Marsiglio et al^[12]^
6	60/F	Nivolumab	Metastatic CCA	392 d	NA	NA	Marsiglio et al^[12]^
7	68/F	Nivolumab 3 mg/kg every 3 wk	Melanoma	60 wk	GAD-, IA2-, ZnT8-, ICA-, Inulin-	DRB1^※^09:01	Sakaguchi et al^[13]^
8	55/F	Nivolumab 2 mg/kg every 3 wk	Melanoma	12 mo	GAD-, IA2-, ZnT8-, ICA-	DRB1^※^04:05, DQB1^※^04:01	Okamoto et al^[14]^
9	48/M	Nivolumab	Melanoma	484 d	GAD-	NA	Ishiguro et al^[15]^
10	49/F	Nivolumab	Melanoma	373 d	GAD-	NA	Ishiguro et al^[15]^
11	74/M	Nivolumab	Lung cancer	19.6 mo (42 cycles)	GAD 0.18, IA2-	NA	Wei et al^[16]^
12	50/M	Nivolumab 240 mg → 480 mg	Esophageal cancer	24.5 mo (240 mg, 38 cycles and 480 mg, 4 cycles)	GAD-, IA2-, Inulin-	Table 2	Present case

CCA = cholangiocarcinoma, DQB1 = HLA-DQB1 antigen, DRB1 = HLA-DRB1 antigen, F = female, HLA = human leukocyte antigen, ICA = islet cell antibody, M = male, NA = not available, NSCLC = non-small cell lung cancer, RCC = renal cell cancer, ZnT8 = zinc transporter 8.

Our study has some limitations. First, because the serum concentrations of nivolumab were not measured, it is unclear whether higher doses resulted in higher blood concentrations. Second, the differences among cancer types were not adequately assessed when examining the factors that may delay the onset of ICI-T1DM. The number of case reports is also highly skewed due to variability in the timing of nivolumab adoption across cancer types. There were more case reports of cancers in which nivolumab was adopted early and fewer where it was introduced more recently. Therefore, it is difficult to compare the differences in the timing of ICI-T1DM onset by cancer type using only currently available case reports.

## 4. Conclusion

In this report, we discussed the potential role of high-dose nivolumab in the development of ICI-T1DM. As nivolumab has demonstrated antitumor effects even at low doses,^[17]^ continuing treatment at lower doses, when feasible, may help reduce immune-related adverse events.

## Author contributions

**Conceptualization:** Sayaka Mabuchi, Atsushi Nagasawa, Satoshi Nabika.

**Data curation:** Sayaka Mabuchi, Naoko Adachi, Atsushi Nagasawa, Satoshi Nabika.

**Formal analysis:** Sayaka Mabuchi.

**Investigation:** Sayaka Mabuchi, Naoko Adachi, Atsushi Nagasawa, Satoshi Nabika.

**Methodology:** Sayaka Mabuchi, Naoko Adachi, Atsushi Nagasawa, Satoshi Nabika.

**Project administration:** Sayaka Mabuchi, Naoko Adachi, Atsushi Nagasawa, Satoshi Nabika.

**Resources:** Sayaka Mabuchi, Naoko Adachi, Atsushi Nagasawa, Satoshi Nabika.

**Supervision:** Naoko Adachi, Atsushi Nagasawa, Satoshi Nabika.

**Validation:** Naoko Adachi, Atsushi Nagasawa, Satoshi Nabika.

**Visualization:** Sayaka Mabuchi, Naoko Adachi, Atsushi Nagasawa.

**Writing – original draft:** Sayaka Mabuchi.

**Writing – review & editing:** Sayaka Mabuchi.
